# Regulatory evaluation of biosimilars throughout their product life-cycle

**DOI:** 10.2471/BLT.17.206284

**Published:** 2018-02-28

**Authors:** Hye-Na Kang, Ivana Knezevic

**Affiliations:** a Essential Medicines and Health Products, World Health Organization, 20 Avenue Appia, 1211 Geneva 27, Switzerland.

## Abstract

The World Health Assembly in 2014 adopted a resolution that recognized the importance of increasing access to biotherapeutic products, of improving their affordability and of ensuring their quality, safety and efficacy. Biosimilars are biotherapeutic products similar to already licensed reference products and are usually developed after patents on the original products have expired. Their introduction into the market is likely to reduce the costs of medicines substantially, thereby improving the availability of treatment for patients. However, there are barriers to market access for biosimilars. This article discusses the factors that give rise to these barriers and explains the importance of regulatory oversight throughout the product life-cycle of biosimilars. The paper also describes the role regulators can play in increasing confidence in biosimilars use by: (i) establishing regulatory oversight of biosimilars throughout their life-cycle, from development to post-licensing oversight, and ensuring that only high-quality, safe and efficacious biosimilars are available on the market; (ii) ensuring regulatory authorities have adequate capacity to assess and monitor the quality, safety and efficacy of biosimilars throughout their life-cycle; and (iii) monitoring the use of biosimilars in public health systems in collaboration with other stakeholders.

## Résumé

En 2014, l'Assemblée mondiale de la Santé a adopté une résolution qui reconnaissait l'importance d'améliorer l'accès aux produits biothérapeutiques, de les rendre moins coûteux et de garantir leur qualité, innocuité et efficacité. Les biosimilaires sont des produits biothérapeutiques similaires à des produits de référence déjà autorisés, qui sont généralement développés après expiration des brevets protégeant les produits originaux. Leur introduction sur le marché a le potentiel de réduire considérablement les coûts des médicaments, améliorant ainsi la disponibilité des traitements pour les patients. Mais certaines barrières font encore obstacle à l’entrée sur le marché des biosimilaires. Cet article évoque les facteurs qui créent ces barrières et explique l'importance d'une supervision réglementaire sur tout le cycle de vie des produits biosimilaires. Cet article décrit également le rôle que les organismes de réglementation pourraient jouer pour améliorer la confiance à l'égard des biosimilaires, notamment: (i) en établissant une supervision réglementaire des biosimilaires sur tout leur cycle de vie, depuis leur développement jusqu'à la surveillance postérieure à leur autorisation de mise sur le marché et en s'assurant que seuls des biosimilaires de grande qualité, sûrs et efficaces sont commercialisés; (ii) en veillant à ce que les autorités de réglementation aient la capacité suffisante pour évaluer et contrôler la qualité, l'innocuité et l'efficacité des biosimilaires pendant tout leur cycle de vie; et (iii) en surveillant l'utilisation des biosimilaires dans les systèmes publics de santé, en collaboration avec d'autres parties prenantes.

## Resumen

La Asamblea de la Organización Mundial de la Salud en 2014 adoptó una resolución que reconoce la importancia de aumentar el acceso a productos bioterapéuticos, de mejorar su asequibilidad y asegurar su calidad, seguridad y eficacia. Los medicamentos biosimilares son productos bioterapéuticos similares a productos de referencia con licencia y usualmente se desarrollan después de que las patentes de los productos originales ya hayan expirado. Su introducción al mercado puede reducir sustancialmente los costes de los medicamentos, por lo tanto, mejora la disponibilidad del tratamiento para los pacientes. Sin embargo, existen barreras de acceso al mercado para los biosimilares. Este articulo trata los factores que dan lugar a esas barreras y explica la importancia de una supervisión reguladora durante el ciclo de vida de los biosimilares. El documento también describe el papel que pueden tener los reguladores para aumentar la confianza en los biosimilares de la siguiente forma: (I) estableciendo supervisiones reguladoras de biosimilares durante su ciclo de vida, desde el desarrollo hasta la supervisión post licencia, y asegurando que solamente biosimilares de alta calidad, seguros y eficaces estén disponibles en el mercado; (ii) asegurando que las autoridades reguladoras tengan la capacidad adecuada para evaluar y controlando la calidad, la seguridad y la eficacia de los biosimilares durante su ciclo de vida; y (iii) controlando el uso de biosimilares en los sistemas de salud públicos en colaboración con otras partes interesadas.

## ملخص

اعتمدت جمعية ****الصحة ****العالمية عام 2014 قرارًا يقر بأهمية زيادة إمكانية الحصول على منتجات العلاجات الحيوية، وتوفيرها بأسعار أكثر معقولية، وضمان جودة هذه المنتجات وسلامتها وفعاليتها. ****وتعد البدائل الحيوية منتجات علاجات حيوية مشابهة لمنتجات مرجعية مرخصة بالفعل، ويتم تطويرها عادةً بعد انتهاء براءات الاختراع المسجلة للمنتج الأصلي. ****ومن المرجح أن يسهم طرح هذه المنتجات في السوق في تخفيض تكاليف الأدوية بشكل كبير مما يؤدي إلى زيادة توافر العلاج للمرضى. ****إلا أنه ثمة عقبات تعوق وصول البدائل الحيوية للسوق. ****وتناقش هذه المقالة العوامل التي تتسبب في ظهور هذه العقبات وتوضح أهمية الإشراف التنظيمي طوال دورة حياة منتجات البدائل الحيوية. ****ويصف التقرير كذلك الدور الذي يمكن أن يلعبه المنظمون لزيادة الثقة في استخدام البدائل الحيوية، وذلك من خلال القيام بالأمور التالية: ** ( **أ( فرض إشراف تنظيمي على البدائل الحيوية طوال دورة حياتها، وذلك بدءًا من مرحلة التطوير إلى الإشراف بعد الترخيص، والتأكد من أن المنتجات المتاحة في السوق هي فقط المنتجات عالية الجودة والمنتجات الآمنة والفعالة؛ (ب) وضمان امتلاك السلطات التنظيمية للقدرة الكافية لمراقبة جودة وسلامة وفعالية البدائل الحيوية طوال دورة حياتها؛ (جـ) ومراقبة استخدام البدائل الحيوية في أنظمة الصحة العامة بالتعاون مع الجهات المعنية الأخرى.

## 摘要

2014 年，世界卫生大会通过了一项决议，承认增加获得生物治疗产品、提高其可购性和确保其质量、安全和功效的重要性。生物仿制药是与已许可的上市参照药品相似的生物治疗产品，通常在原产品的专利过期后开发。将它们引入市场，可能会大幅降低药物的成本，从而提高病人治疗的有效性。然而，生物仿制药在市场准入方面存在障碍。本文讨论了造成这些障碍的因素，并说明了在生物仿制药的产品寿命周期中监管监督的重要性。本文还描述了监管部门在提高对生物仿制药使用的信心方面所发挥的作用：(i) 在从开发到许可后的监督整个寿命周期内建立对生物仿制药的监管监督，并确保市场上只有高质量、安全和有效的生物仿制药；(ii) 确保监管当局有足够的能力评估和监测生物仿制药在其整个寿命周期内的质量、安全性和有效性；(iii) 与其他利益攸关方合作，监测公共卫生系统中生物仿制药的使用情况。

## Резюме

Всемирная ассамблея здравоохранения в 2014 году приняла резолюцию, в которой признается важность расширения доступа к биотерапевтическим препаратам, повышения их ценовой доступности и обеспечения их качества, безопасности и эффективности. Биоаналоги — это биотерапевтические препараты, которые аналогичны лицензированным референтным препаратам и обычно разрабатываются после истечения срока действия патентов на оригинальные продукты. Их внедрение на рынок должно значительно снизить стоимость лекарственных препаратов, тем самым улучшив доступность лечения для пациентов. Однако существуют препятствия для внедрения биоаналогов на рынок. В этой статье обсуждаются факторы, которые создают эти барьеры, и объясняется важность надзора со стороны контролирующих органов за биоаналогами на протяжении всего их жизненного цикла. В документе также описывается роль регулирующих государственных органов в повышении доверия к использованию биоаналогов с помощью следующих мероприятий: (i) установление надзора со стороны контролирующих органов за биоаналогами на протяжении всего их жизненного цикла, от разработки до постлицензионного контроля, и обеспечение того, чтобы на рынке были доступны только высококачественные, безопасные и эффективные биоаналоги; (ii) обеспечение того, чтобы регулирующие органы имели достаточный потенциал для оценки и контроля качества, безопасности и эффективности биоаналогов на протяжении всего их жизненного цикла; и (iii) мониторинг использования биоаналогов в системах общественного здравоохранения в сотрудничестве с другими заинтересованными сторонами.

## Introduction

Countries around the world face the common problems of an aging population and the associated increase in the prevalence of chronic diseases. The success of biotherapeutic products, such as such as large complex proteins, for treating human diseases, in the treatment of many life-threatening chronic conditions, combined with the approaching expiry of patent protection on these products, has led to increased interest in the development of biosimilars, which are products that are similar to the originals. The patents of many best-selling biotherapeutic products have already expired or will soon reach their expiry date. For example, the patent on the breast cancer drug Herceptin, a monoclonal antibody with international nonproprietary name trastuzumab, expired in July 2014 in the European Union and will expire in June 2019 in the United States of America.^1^ Currently, several companies worldwide have developed biosimilar versions of trastuzumab. The development of biosimilars after the expiry of patents on the original products is expected to make biotherapeutics available at more affordable prices and to increase their use by providing more treatment options. European Union countries have the longest history of using biosimilars and it is expected that, as a result, these countries’ health-care systems could save 11.8 to 33.4 billion euros between 2007 and 2020.^2^ A recent report concluded that competition from biosimilars has led to a consistent reduction in the average price of treatment in clinical areas where they have been introduced and that, in some countries, patients began to have access to product classes that were previously unavailable.^3^

In its guidelines on the evaluation of similar biotherapeutic products, the World Health Organization (WHO) defines a similar biotherapeutic product (also called a biosimilar) as a “biotherapeutic product that is similar in terms of quality, safety and efficacy to an already licensed reference product”.^4^ Examples of biosimilars include growth hormone, erythropoietin and monoclonal antibodies for the treatment of a wide range of diseases. 

Biotherapeutic products are generally relatively large and complex entities that are more difficult to characterize than simpler, chemical drugs. They are a heterogeneous group of proteins whose structures are sensitive both to the inherent variability of the protein production, or expression, system and to changes in manufacturing processes. Thus, no biotherapeutic product or biosimilar can be scientifically or technically identical to the originator’s product. Nor can it be identical to the different version of itself that is produced after a change in manufacturing process. In fact, this is also true for different batches of the same product. Consequently, if these biological substances are to be used routinely in clinical practice, it is essential that different production lots are of consistent quality. Therefore establishing robust manufacturing and quality control procedures, many of which may be in-process controls carried out during manufacturing, is needed.^5^

In May 2014, the sixty-seventh World Health Assembly adopted a resolution on access to biotherapeutic products and on ensuring their quality, safety and efficacy.^6^ Since then, action has been taken to help WHO Member States to increase their expertise in evaluating biosimilars and expand their capacity to do so; to improve regulatory convergence; and to use existing resources more effectively. As part of its biological standardization programme, WHO provides written guidelines on the evaluation of biological products (including biosimilars) to ensure their quality, safety and efficacy.^4^^,^^7^^–^^9^ These guidelines are usually incorporated into national requirements to ensure that the products produced and used in a country conform to current international standards, such standards are published as recommendations in WHO’s Technical Report Series. In addition, regulatory guidance documents produced by WHO also provide advice for national regulatory authorities and manufacturers on the evaluation of biological products, with the aim of establishing a harmonized regulatory framework for products available on international markets. 

The current regulatory framework for biosimilars has been successful in enabling many biosimilar products to gain regulatory approval around the world. However, there are barriers to market access for biosimilars. One is the belief of many clinicians that a similar biotherapeutic product cannot be as good as the original for their patients. More precisely, there is a lack of understanding that biosimilars have undergone comparability studies that demonstrate their similarity, that data on the original products can be extrapolated and that biosimilars become independent products after licensing and undergo their own development. This article discusses the factors that give rise to these barriers and explains the role of regulators and the importance of regulatory oversight throughout the product life-cycle of biosimilars, both of which are critical for increasing confidence in their use. In addition, WHO’s role in improving regulatory convergence at the global level by developing standards and helping ensure these standards are incorporated into national regulatory requirements is explained briefly.

## Life-cycle and regulatory evaluation

Like other biological medicines, biosimilars have a product life-cycle, which starts with research and development and continues through manufacturing to regulatory evaluation of quality, safety and efficacy for both licensing and post-licensing oversight. However, the life-cycle of a biosimilar is unique in the sense that its regulatory approval relies on the safety and efficacy data and knowledge gained during the development and licensing of an originator, or reference, product. However, once licensed, the biosimilar becomes an individual product and post-licensing evaluation should be carried out as for any other biological product.^7^

The safety and efficacy of a biosimilar is established by demonstrating its similarity to a reference product.^4^ The concept of extrapolating data that is used for the licensure of biosimilars is not new to regulators or manufacturers, it is an established scientific and regulatory principle that has been exercised for many years, for example, in considering changes to manufacturing processes of the originator biologicals.^7^^,^^10^ Regulators have learned how much variation is acceptable between different versions of a product from their experience with postapproval manufacturing changes. Prescribers, such as physicians and clinicians, tend to judge the safety and efficacy of medicines using clinical trial data. Although clinical data is important, it is a mistake to overlook the extensive data on the characteristics of a medicine derived using the state-of-the-art, comparative, analytical methods that underlie biosimilar development programmes.^10^ Usually, analytical assessments are more sensitive for detecting differences between, or changes in, products than the endpoints used in clinical trials (i.e. the clinical outcomes measured objectively to determine whether the intervention is beneficial or not). Thus, a biosimilar with chemical, physical and biological attributes that are highly similar to those of the reference product would be expected to have the same pharmacological characteristics as the reference product and a similar safety and efficacy profile for every clinical indication.

Manufacturing processes are often altered after regulatory approval and medicines can undergo changes during their product life-cycle. Reasons for these changes include: improvements in the manufacturing process; an increase in the scale of production; movement to a new manufacturing site; improvements in product stability; and the need to comply with new regulatory requirements.^7^^,^^8^ Such changes are welcome as they often represent improvements. However, a minor alteration in the manufacturing process may have a large impact on the final product, which could, for example, lead to serious adverse events in patients. For instance, a change in the formulation of erythropoietin resulted in an increase in the occurrence of pure red cell aplasia.^11^ Thus, any change in the manufacturing processes of a licensed medicine, particularly a biological, should be approved by the regulators once the medicines produced before and after the manufacturing change have been shown to be comparable in a comparability assessment.^7^ Although the general scientific principles of comparability assessments following changes in manufacturing processes are applicable to comparability assessments for biosimilars, more extensive and comprehensive data are required for biosimilarity assessment. Once a biosimilar has been approved, there is no regulatory requirement for its biosimilarity to the reference product to be demonstrated again at any other time, the biosimilar effectively becomes a stand-alone product.^7^ After approval, therefore, the management of biosimilars throughout their life-cycle is the same as for other biologicals and, accordingly, biosimilars should be managed in a way that ensures their benefits outweigh their risks throughout their life-cycle. In some cases, specific safety monitoring requirements that have been imposed on the reference product or product class should be incorporated into the biosimilar pharmacovigilance plan, unless there is compelling evidence not to do so.^4^ Recently, at the request of Member States, WHO has published new guidelines on postapproval changes to biotherapeutic products.^7^^,^^12^ In general, the guiding principles that apply to postapproval changes to biotherapeutic products also apply to postapproval changes to biosimilars.

Some countries have biotherapeutics on their markets that are claimed to be copies of original products (i.e. so-called non-innovator or copy-version products). These medicines have not been approved through a biosimilar approval procedure but have, instead, been licensed as generics or small-molecule medicines.^9^ However, as biotherapeutics are relatively large and complex proteins, procedures established for generics or small-molecule medicines are not suitable for the development, evaluation or licensing of biosimilars.^4^ As stated in WHO’s guidelines on the evaluation of similar biotherapeutic products, a biosimilar that has not been demonstrated to be similar to a reference product through head-to-head comparisons should not be described as similar or be called a biosimilar.^4^ These guidelines also stipulate that, “regular review of NRAs [national regulatory authorities] for their licensing, for adequacy of their regulations for providing oversight, and for the processes and policies that constitute the regulatory framework is an essential component of a well-functioning and up-to-date regulatory oversight for biotherapeutics”.^4^ If problems arise with products that were licensed before national regulations for biosimilars had been established, the regulatory authority should take action to identify the problematic products in its market, to assess the risk–benefit balance of their use and to decide whether additional evaluations are needed. Based on the results of these evaluations, some products may be removed from the market, because of concerns about safety or efficacy. This step-wise approach to regulatory assessment is recommended by WHO and is intended to be flexible and to increase access to biotherapeutic products, including biosimilars of assured quality, safety and efficacy.^9^

## Role of regulatory authorities

Market access to biosimilars can be restricted by several of factors: (i) manufacturing processes may be expensive and complex; (ii) patents on the manufacturing processes of the original product may not have expired; (iii) biosimilar manufacturers may have limited access to data on the original product; (iv) appropriate regulatory frameworks may not be in place; and (v) government policies on switching to biosimilars, pricing and reimbursement may be lacking. Experience with the introduction of small-molecule generic medicines showed that gaining the trust of all stakeholders, including policy-makers, regulators, physicians and other health-care providers, is essential for increasing the uptake of biosimilars. Governments should provide a robust regulatory framework; ensure intellectual property rights are respected; guarantee fair pricing; devise a policy on reimbursements and incentives; and ensure health-care professionals and patients are fully informed.^13^ In particular, it is important that other stakeholders understand the role played by regulatory authorities in ensuring better access to biosimilars. Regulators who review and approve biosimilars are in a very good position to provide reassurance about their use.

One of the main barriers to the uptake of biosimilars is the perception that, in general, they may not have been studied thoroughly enough and that, therefore, they may not be safe.^10^ This perception is due to a lack of knowledge about the scientific principles underlying the development and licensing of biosimilars and to the inappropriate labelling of non-innovator and copy-version products as biosimilars. Regulatory authorities should develop a specific, appropriate, regulatory framework for approving biosimilars that is distinct from the regulatory procedures previously applied to copy-version products, where regulatory evaluation was not well-defined. In addition, regulatory authorities should also make an effort to communicate with, and educate, all stakeholders, including patients, about biosimilars and their approval. The publication of public assessment reports on biosimilars and of the relevant regulations and guidelines could provide useful communication tools. The Biosimilar Working Group of the International Pharmaceutical Regulators Programme published a template for biosimilar assessment reports that could be used by regulators worldwide; it is entitled *Public assessment summary information for biosimilars (PASIB).*^14^ The provision of such information will contribute to better transparency and increase public trust in biosimilars.^15^

Regulatory authorities could improve access to biosimilars by increasing the efficiency of their review processes and of regulatory evaluation, for example, by increasing their capacity and reducing the time needed, without compromising the quality of the review process. In addition, they should align national regulatory requirements with WHO’s guiding principles to avoid the need for bridging studies and to reduce development costs. Regulatory authorities, especially in countries with limited regulatory resources, should focus on activities that eliminate duplications of effort and add genuine value. WHO and International conference of drug regulatory authorities recommend that joint, collaborative assessments should be carried out with neighbouring countries, where appropriate, and that efforts should be made to ensure that products that have already undergone rigorous evaluation in other countries are not evaluated again.^12^^,^^15^^,^^16^

In 2017, WHO initiated discussions on a pilot project to prequalify two biosimilar monoclonal antibody products: (i) rituximab, which is principally used to treat non-Hodgkin's lymphoma and chronic lymphocytic leukaemia; and (ii) trastuzumab, which is used to treat breast cancer. The purpose of the pilot study was to explore the possibility of assisting countries with limited expertise and resources to evaluate products such as these, thereby increasing access to biosimilars. Details of this pilot study and of associated issues are available in the report of an expert consultation held in Geneva, Switzerland in May 2017, which aimed to improve access to, and the use of, biosimilars.^13^

## Future of biosimilars

By competing with the originator’s biotherapeutic products, biosimilars provide alternative treatment options, thereby reducing the price of these biotherapeutics and increasing their availability. However, improving access to biosimilars and ensuring they are used appropriately requires a high degree of collaboration between all stakeholders, each of which has a distinct role. The main roles of regulatory authorities, for example, are to provide regulatory oversight of biosimilars throughout their product life-cycle and to ensure that only high-quality, safe and efficacious biosimilars are available on the market.^12^ To achieve this, the capacity of regulatory authorities should be increased.^12^ However, strengthening regulatory systems by increasing capacity will be particularly challenging in resource-limited settings. Regulatory authorities in these settings should consider establishing regulatory procedures that improve the efficiency of the approval process. Approval could be based on a collaborative review carried out with other regulatory authorities or on a previous expert review carried out, for example, when another regulatory authority with the appropriate expertise granted approval. In addition, regulatory authorities should monitor the use of biosimilars in public health systems in collaboration with other stakeholders. To assist, WHO has established global standards to ensure the quality, safety and efficacy of biotherapeutics, including biosimilars, at all stages of their life-cycle.^4^^,^^7^^–^^9^ These standards could serve as a basis for mutual recognition of regulatory oversight and for regulatory convergence at the global level.
